# Detecting Static and Dynamic Differences between Eyes-Closed and Eyes-Open Resting States Using ASL and BOLD fMRI

**DOI:** 10.1371/journal.pone.0121757

**Published:** 2015-03-27

**Authors:** Qihong Zou, Bin-Ke Yuan, Hong Gu, Dongqiang Liu, Danny J. J. Wang, Jia-Hong Gao, Yihong Yang, Yu-Feng Zang

**Affiliations:** 1 Center for MRI Research and Beijing City Key Lab for Medical Physics and Engineering, Peking University, Beijing, China; 2 Neuroimaging Research Branch, National Institute on Drug Abuse, National Institutes of Health, Baltimore, Maryland, United States of America; 3 Center for Cognition and Brain Disorders, Affiliated Hospital, Hangzhou Normal University, Hangzhou, Zhejiang, China; 4 Zhejiang Key Laboratory for Research in Assessment of Cognitive Impairments, Hangzhou Normal University, Hangzhou, Zhejiang, China; 5 Department of Neurology, University of California Los Angeles, Los Angeles, California, United States of America; 6 McGovern Institute for Brain Research, Peking University, Beijing, China; Penn State University, UNITED STATES

## Abstract

Resting-state fMRI studies have increasingly focused on multi-contrast techniques, such as BOLD and ASL imaging. However, these techniques may reveal different aspects of brain activity (e.g., static vs. dynamic), and little is known about the similarity or disparity of these techniques in detecting resting-state brain activity. It is therefore important to assess the static and dynamic characteristics of these fMRI techniques to guide future applications. Here we acquired fMRI data while subjects were in eyes-closed (EC) and eyes-open (EO) states, using both ASL and BOLD techniques, at two research centers (NIDA and HNU). Static brain activity was calculated as voxel-wise mean cerebral blood flow (CBF) using ASL, i.e., CBF-mean, while dynamic activity was measured by the amplitude of low frequency fluctuations (ALFF) of BOLD, i.e., BOLD-ALFF, at both NIDA and HNU, and CBF, i.e., CBF-ALFF, at NIDA. We showed that mean CBF was lower under EC than EO in the primary visual cortex, while BOLD-ALFF was higher under EC in the primary somatosensory cortices extending to the primary auditory cortices and lower in the lateral occipital area. Interestingly, mean CBF and BOLD-ALFF results overlapped at the visual cortex to a very small degree. Importantly, these findings were largely replicated by the HNU dataset. State differences found by CBF-ALFF were located in the primary auditory cortices, which were generally a subset of BOLD-ALFF and showed no spatial overlap with CBF-mean. In conclusion, static brain activity measured by mean CBF and dynamic brain activity measured by BOLD- and CBF-ALFF may reflect different aspects of resting-state brain activity and a combination of ASL and BOLD may provide complementary information on the biophysical and physiological processes of the brain.

## Introduction

Blood oxygenation level dependent (BOLD) functional magnetic resonance imaging (fMRI) is a noninvasive neuroimaging technique that is widely used due to its relatively high sensitivity, ease of implementation, and good spatial and temporal resolution. Resting-state BOLD fMRI has been increasingly adopted to assess the functional connectivity of brain networks following the seminal finding that BOLD signals are temporally synchronized in the sensorimotor system [1]. Though spontaneous BOLD fluctuations during the resting state have been demonstrated to reflect neuronal activity [2–5], BOLD is an indirect measure of neuronal activity, as it depends on blood oxygenation, which is the combined response of CBF, cerebral metabolic rate of oxygen (CMRO_2_), and cerebral blood volume. In contrast to BOLD, arterial spin labeling (ASL) fMRI can quantify regional CBF, a single physiological parameter closely related to cerebral metabolism and neuronal activity [6–8]. Recently, attention has been attracted to the combination and comparison of resting-state BOLD and ASL signals. Previous studies have demonstrated the coupling of CBF with resting-state brain activity derived from BOLD, providing evidence that intrinsic BOLD activity has a physiological basis [9–11]. As of yet, however, no study has directly compared the between-condition results yielded by these two different fMRI techniques.

Most ASL studies measure the mean CBF (referred to as CBF-mean hereafter) over a scanning session at the voxel level. CBF-mean could be considered as an index reflecting static brain activity over the duration of scanning. In contrast, the amplitude of low-frequency fluctuations (ALFF) of BOLD (referred to as BOLD-ALFF hereafter) measures the variation over time or dynamic fluctuations of brain activity at the voxel level [12, 13] and has been used to detect altered brain activities in many brain disorders [13–19]. Similarly, spontaneous fluctuations of CBF could be captured [20] by ALFF of CBF (named CBF-ALFF below), which has been used for voxelwise measurement of the dynamic characteristics of resting-state perfusion signals. To keep the terms consistent with those used in our previous study [20], BOLD-ALFF and CBF-ALFF refer to dynamic measurements and CBF-mean refers to a static measurement of the brain activity in this study.

Using both ASL and BOLD techniques, we acquired data from subjects in eyes-closed (EC) and eyes-open (EO) states, two physiological states with different levels of brain activity [21, 22]. We collected BOLD and ASL data at two independent research centers for validation purposes and aimed to investigate: 1) whether static and dynamic state differences between EC and EO could be detected by BOLD and ASL fMRI; 2) if yes, whether static and dynamic state differences detected would show spatial overlap; and 3) whether the static and dynamic state differences could be cross-validated by the two research centers. Both BOLD and ASL fMRI measure hemodynamic responses induced by neuronal activity. However, mean CBF captures static characteristics of resting-sate brain, while dynamics measured by ALFF reflect variance in resting fluctuations. Moreover, state differences have been shown mainly in the primary motor system and auditory cortex by BOLD-ALFF [23, 24], while in the visual cortex by mean CBF [25] in separate studies. Thus, we hypothesized that convergent and divergent state differences between EC and EO would be detected by static and dynamic brain activity.

## Materials and Methods

### Subjects

#### Dataset 1

Forty-eight healthy control subjects (27.4 ± 7.1 years old, 25 females) participated in the study. They were recruited under a protocol approved by the Institutional Review Board of the Intramural Research Program of the National Institute on Drug Abuse (NIDA), NIH.

#### Dataset 2

Thirty-four healthy control subjects (23.7 ± 1.9 years old, 18 females) participated in the study. All experiments were approved by the ethics committee of the Center for Cognition and Brain Disorders, Hangzhou Normal University (HNU).

All subjects were screened with a questionnaire to ensure they had no history of neurological illness or psychiatric disorders. Signed informed consent was obtained from all subjects prior to study enrollment.

### Experimental paradigm

All subjects underwent four resting-state scans, during which they were asked to relax with either EC or EO. BOLD and ASL data, respectively, were acquired during the two resting states. The order of the two resting states and the two fMRI techniques was counter-balanced across subjects. No other task-based fMRI scans were performed before the resting-state scans. At NIDA, working memory task scans were acquired after these resting-state scans [10, 26].

### Data acquisition

#### Dataset 1

MRI data were collected on a 3-T Siemens Allegra MR scanner (Siemens, Erlangen, Germany) equipped with a quadrature volume head coil at NIDA, NIH. Head movement was minimized using foam padding custom-made for each subject.

A pseudo continuous ASL (pCASL) technique [27, 28] was adopted for CBF measurement. Interleaved control and label images were acquired using a gradient echo EPI sequence with the following parameters: TR/TE = 4500/21 ms, FA = 90°, slice thickness = 5 mm with 20% gap, 20 slices, FOV = 220×220 mm^2^ with in-plane resolution of 3.44×3.44 mm^2^, labeling duration = 1600 ms, label offset = 80 mm, post-labeling delay = 1200 ms and bipolar crusher gradient = 9 sec/mm^2^ for suppression of signals in the intraarterial spins [29]. The duration of the resting-state scan was 7.5 minutes, and it included 50 pairs of control and label images.

A single-shot gradient echo EPI sequence was used to acquire BOLD data. The imaging parameters were as follows: TR/TE = 2000/27 ms, FA = 77°, 39 slices, thickness/gap = 4/0 mm, FOV = 220×220 mm^2^ with in-plane resolution of 3.44×3.44 mm^2^. The duration of the resting-state scan was 8 minutes, and it included 240 images.

For registration purposes, high-resolution anatomical images were acquired from each subject using a 3-D magnetization-prepared rapid gradient echo T1-weighted sequence (256×192×160 matrix size; 1×1×1 mm^3^ in-plane resolution; TI/TR/TE = 1000/2500/4.38 ms; flip angle = 8°).

#### Dataset 2

MRI images were acquired using a GE healthcare MR-750 3-T scanner (GE Medical Systems, Milwaukee, WI) with an eight-channel head coil at the Center for Cognition and Brain Disorders of HNU. The subjects lay supine with their heads snugly fixed by straps and foam pads to minimize head movement.

The CBF images were acquired with a 3D pCASL sequence that uses a fast spin echo acquisition with background suppression [28]. The imaging parameters were as follows: TR/TE = 4781/11.1 ms, spiral readout = 12 arms × 512 samples, 42 × 3.0 mm^2^ axial sections, voxel size = 1.7 × 1.7 × 3 mm^3^, number of excitation (NEX) = 3, labeling duration = 1500 ms, and post-labeling delay = 1525 ms. The duration of the resting-state scan was 6 minutes and 48 seconds.

The BOLD images were acquired using a gradient echo EPI pulse sequence with the following parameters: TR/TE = 2000/30 ms, flip angle = 60°, 37 slices, thickness/gap = 3.4/0 mm, FOV = 220×220 mm^2^ with an in-plane resolution of 3.44×3.44 mm^2^. The duration of the resting-state scan was 8 minutes, and it included 240 images.

A high-resolution 3D volume was acquired using a spoiled gradient-recalled pulse sequence (176 sagittal slices, thickness = 1 mm, TR/TE = 8100/3.1 ms, flip angle = 9°, FOV = 250×250 mm^2^).

### Data processing

Both the resting-state (EC and EO) pCASL and BOLD data were preprocessed using the Analysis of Functional Neuroimages (AFNI) software package [30].

For pCASL data acquired at NIDA, control and label images with the least deviation from the trend of the respective time courses were chosen as a base for motion correction and were linearly registered to each other. Then, control and label images were registered to their corresponding bases, separately. Slice time correction was not performed for pCASL data. Following head motion correction, images were spatially smoothed with an 8-mm Gaussian kernel. To reduce potential BOLD contamination, CBF-weighted time series were created by subtraction of the time-matched control and label images using sinc interpolation [31, 32]. The absolute CBF time series was calculated using a one-compartment model [33] as follows:

$$f=\frac{\lambda \text{Δ}M_{ts}R_{1a}}{2\alpha M_{con}\{\text{exp}(-wR_{1a})-\text{exp}[-(\tau +w)R_{1a}]\}}$$ (1)

where *λ* (0.9 ml/g) is the blood-tissue water partition coefficient, Δ*M*_*ts*_ is the CBF-weighted time series, *R*_1*a*_ (0.67 sec^-1^) is the longitudinal relaxation rate of blood, *α* (80%) is the tagging efficiency, *M*_*con*_ is the average intensity of control images, *w* (1200 ms) is the post-labeling delay time, and *τ* (1600 ms) is the duration of the labeling pulse. Then, the CBF time series were averaged together to obtain absolute CBF-mean maps. For pCASL data acquired at HNU, CBF-mean quantification was conducted with similar processing strategies using Functool (version 9.4.04), an automated image post-processing tool implemented in the GE healthcare MR-750 system. Spatial smoothing with an 8-mm Gaussian kernel was performed after quantitative CBF-mean maps were obtained.

For BOLD data acquired at both NIDA and HNU, the same processing strategies were employed. Preprocessing steps included slice-timing correction, head motion correction, linear trend removal, and spatial smoothing with an 8-mm Gaussian kernel. We calculated voxel-wise BOLD-ALFF over a frequency range of 0.01–0.1 Hz. Then, the BOLD-ALFF maps were transformed into *z*-score maps by subtracting the mean for the entire brain from each voxel, and dividing by the standard deviation for the whole brain [12, 34].

A similar procedure was performed to calculate the ALFF of CBF-weighted time series (CBF-ALFF), preprocessed as described above for the NIDA dataset. As temporal resolution of the CBF-weighted time courses were at an effective TR of 9 sec, the CBF-ALFF analysis was conducted over a frequency range of 0–0.055Hz. The HNU ASL data was not available for ALFF analysis, as no time courses were provided.

Finally, the resting-state CBF-mean and BOLD-ALFF maps were spatially normalized to Talairach and Tournoux (TT) space with a resampling resolution of 3×3×3 mm^3^ to facilitate group analysis.

### Statistical analyses

Brain activity differences between EC and EO were obtained by paired *t*-tests of BOLD-ALFF, CBF-mean (quantitative), and CBF-ALFF data. Significance thresholds for the paired *t*-tests were set at corrected *p* < 0.05 based on Monte Carlo simulations to correct for multiple comparisons over the whole brain. The corrected threshold corresponded to a single voxel’s *p* < 0.01 and a minimum cluster size of 3996 mm^3^. The same threshold was adopted for the two independent datasets.

## Results

### State differences between EC and EO using BOLD and CBF data from NIDA

**Fig 1:**
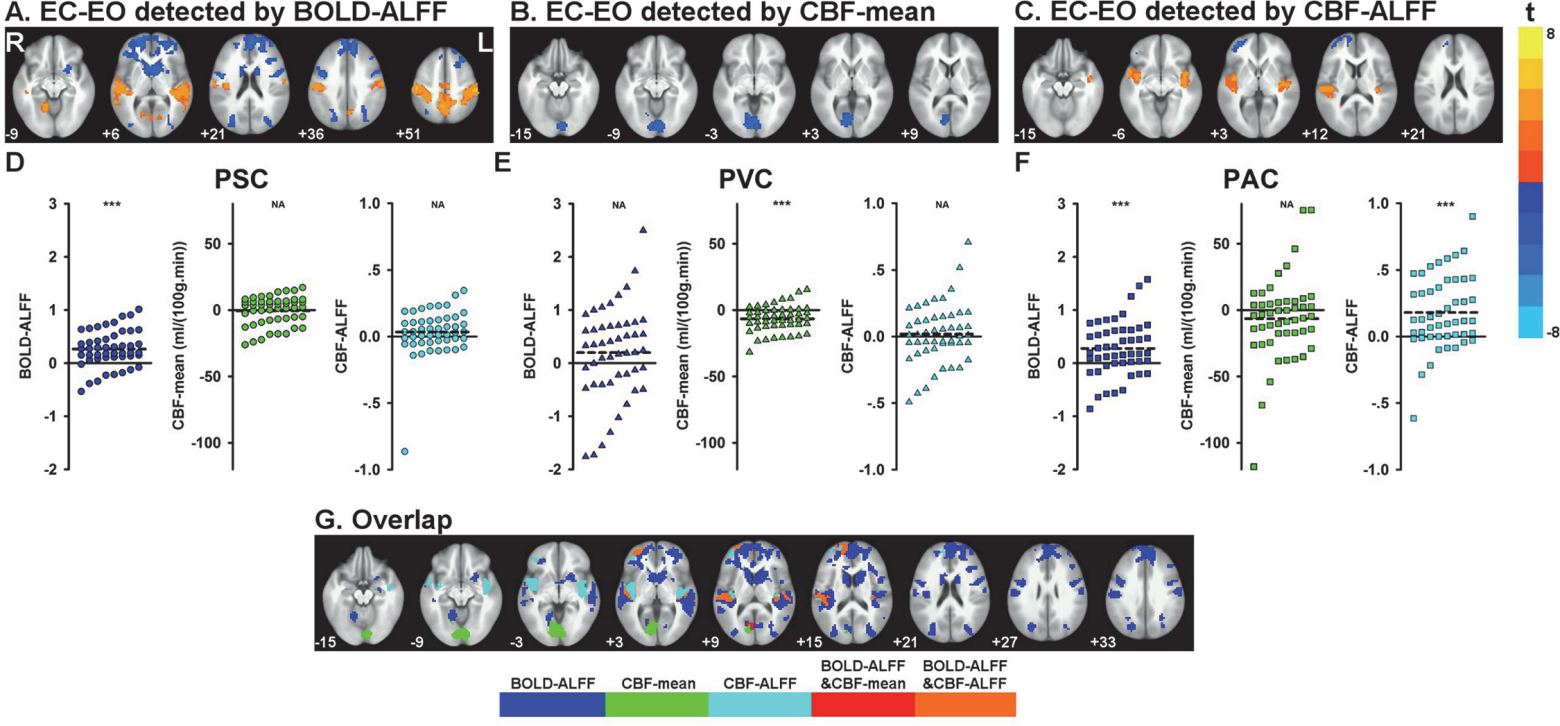
BOLD-ALFF (A), CBF-mean (B), and CBF-ALFF (C) for EC compared to EO using NIDA data. The delta (i.e., EC minus EO) of BOLD-ALFF (D), CBF-mean (E) and CBF-ALFF (F) of three typical voxels from each subject is shown (PSC: primary somatosensory cortex, with peak *t* value at [–44, 22,41] in Fig. 1A; PVC: primary visual cortex, with peak *t* value at [–8, 79, –4] in Fig. 1B; PAC: primary auditory cortex, with peak t value at [–44, 22,8] in Fig. 1C). Spatial overlap of regions detected by both BOLD-ALFF (regions labeled in blue) and CBF-mean (regions labeled in green) are labeled in red, and by both BOLD- and CBF-ALFF (regions labeled in cyan) are labeled in orange (G). No spatial overlap was seen between regions detected by CBF-mean and CBF-ALFF, or among BOLD-ALFF, CBF-mean and CBF-ALFF.

Using the NIDA data, BOLD-ALFF was found to be significantly higher for EC than EO in the primary somatosensory cortices (PSC), primary auditory cortices (PAC), supplementary motor area (SMA), and precuneus, and significantly lower for EC than EO in the lateral occipital cortex, medial and lateral frontal cortex near the frontal pole, caudate, and premotor area (Fig. 1A). CBF-mean in the primary visual cortex (PVC) was lower for EC than EO (Fig. 1B). CBF-ALFF was higher in the bilateral PAC under EC than EO and lower in the left lateral frontal cortex (Fig. 1C). To illustrate the differences between EO and EC, three peak voxels in the PSC [–44, 22, 41], PVC [–8, 79, –4] and PAC [–44, 22,8] were selected from the paired *t*-maps of BOLD-ALFF, CBF-mean and CBF-ALFF, respectively. Higher BOLD-ALFF was seen in the PSC (39 out of 48 subjects) (Fig. 1D) and PAC (36 out of 48 subjects) (Fig. 1F) for EC than for EO, while lower CBF-mean was seen in the PVC (36 out of 48 subjects) (Fig. 1E), and higher CBF-ALFF in the PAC (36 out of 48 subjects) (Fig. 1F). In order to view the overlap, we merged the thresholded binary maps of the paired *t*-test results of BOLD-ALFF, CBF-mean and CBF-ALFF. As shown in Fig. 1G, the two sets of brain regions detected by CBF-mean and BOLD-ALFF overlapped at the PVC by only 29 voxels, and regions detected by CBF-ALFF were generally a subset of those detected by BOLD-ALFF. No spatial overlap between CBF-mean and CBF-ALFF was detected. The overlapping regions detected by CBF-ALFF and BOLD-ALFF were apparently much larger than those detected by CBF-mean and BOLD-ALFF, and those detected by CBF-mean and CBF-ALFF.

### Validation of the main findings using HNU data

**Fig 2:**
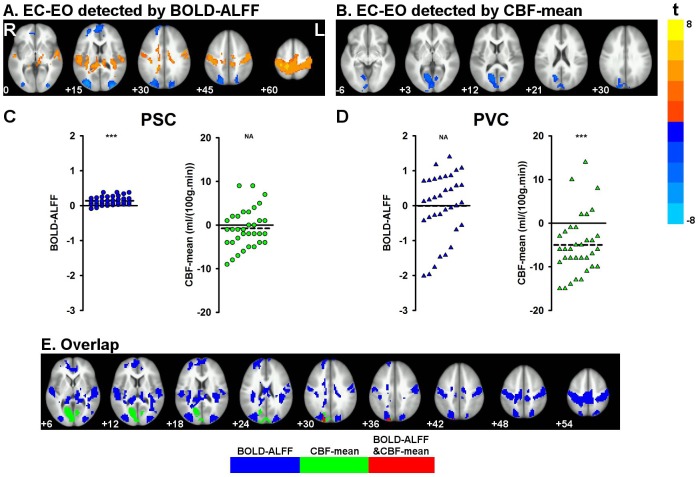
BOLD-ALFF (A) and CBF-mean (B) for EC compared with EO using HNU data. The delta (i.e., EC minus EO) of BOLD-ALFF (C) and CBF-mean (D) of two typical voxels from each subject is shown (PSC: primary somatosensory cortex, with peak *t* value at [–17, 34,53] in Fig. 2A; PVC: primary visual cortex, with peak *t* value at [–11, 64,5] in Fig. 2B). Spatial overlap between the brain regions that showed significant state differences detected by BOLD-ALFF (regions labeled in blue) and CBF-mean (regions labeled in green) with HNU data (E), which are labeled in red.

Similar state differences were observed in the HNU data. Higher BOLD-ALFF for EC than EO was observed in the PSC, PAC, SMA, and precuneus, while lower BOLD-ALFF for EC than EO was observed in the lateral occipital cortex, and medial and lateral frontal cortex (Fig. 2A). CBF-mean in the PVC was lower for EC than EO (Fig. 2B). Two peak voxels in the PSC [–17, 34,53] and the PVC [–11, 64,5] were selected from the paired *t*-maps of BOLD-ALFF and CBF-mean, respectively. Higher BOLD-ALFF was shown in the PSC (31 out of 34 subjects) for EC than for EO (Fig. 2C), while a lower CBF-mean was shown in PVC (28 out of 34 subjects) (Fig. 2D). A small cluster with only 32 voxels at the right middle occipital gyrus showed overlapping between-state differences when using BOLD-ALFF and CBF-mean of the HNU data (Fig. 2E).

**Fig 3:**
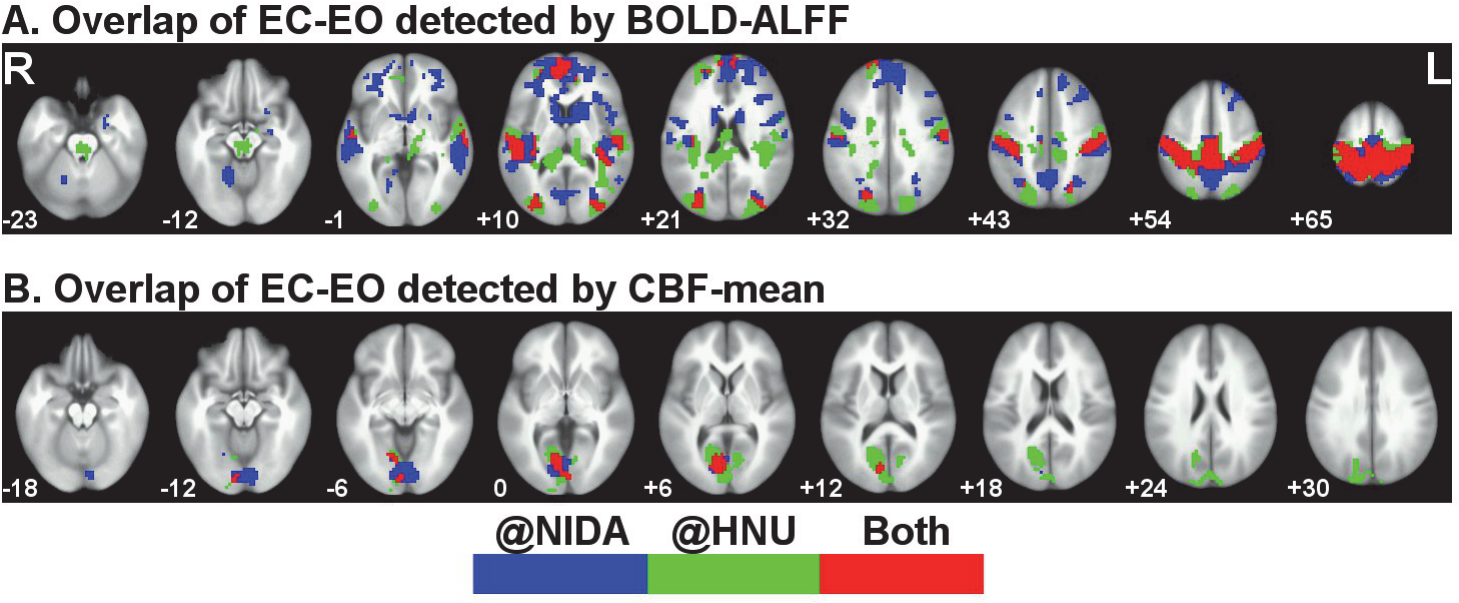
Spatial overlap of paired *t*-maps from NIDA and HNU using BOLD-ALFF (A) and CBF-mean (B). Significant regions detected in the data acquired at NIDA are labeled in blue, and those detected in the data acquired at HNU are labeled in green. Spatial overlap of regions detected by both research centers is labeled in red.

To investigate convergence across research sites, we overlapped the results from the HNU data with those from the NIDA data. For the EC-EO results using BOLD-ALFF, the PSC, PAC, SMA, medial and lateral frontal cortex, and lateral occipital cortex showed consistent between-state differences across the two research sites (Fig. 1A, Fig. 2A, and Fig. 3A). Both the NIDA and HNU datasets showed higher CBF-mean for EO than for EC in the PVC (Fig. 1B, Fig. 2B, and Fig. 3B), with some overlap.

## Discussion

We observed remarkable state differences between EC and EO using BOLD-ALFF (i.e., BOLD dynamics) and CBF-mean (i.e., static CBF) to examine data from the same groups of subjects across two independent research centers. BOLD-ALFF was consistently higher for EC than for EO in the PSC, PAC, and SMA, and lower for EC than EO in the lateral occipital cortex and frontal cortices. CBF-mean, measured by ASL fMRI, was consistently higher for EO than EC in the PVC. Convergent state differences detected by both BOLD and ASL fMRI were located in only a small volume of the visual cortex. These BOLD-ALFF and CBF-mean results were highly consistent in the two independent datasets (NIDA and HNU). For the NIDA data, CBF-ALFF (i.e., CBF dynamics) was higher in the bilateral PAC and lower in the lateral frontal cortex under EC than EO. The brain areas detected by CBF-ALFF showed no spatial overlap with those detected by CBF-mean, which was generally a subset of regions detected by BOLD-ALFF.

### Static and dynamic state differences between EC and EO using BOLD and CBF

Although previous resting-state fMRI studies have compared EC and EO, using either BOLD or ASL separately, the current study combined BOLD and ASL to reveal differences between EC and EO. Further, we validated our multi-contrast state differences with two independent datasets.

In general, our findings of state differences between EC and EO using BOLD-ALFF were consistent with previous observations [23, 24, 35]. Liu and colleagues [23] performed split-half validation of the ALFF difference between EO and EC. They found high reproducibility of the higher ALFF for EC than EO in the PSC, PAC, SMA, as well as high reproducibility of the lower ALFF for EC than EO in the lateral occipital cortex. The current BOLD-ALFF results from two independent datasets demonstrated that those ALFF differences were highly reproducible across research centers, expanding previous reproducibility findings using split-half validation [23].

Higher amplitude of BOLD fluctuations in the visual cortex under EC than EO have been reported [36–40], which were inconsistent with our findings. One potential explanation for this inconsistency was whether or not spatial z-score normalization was performed on the strength of BOLD dynamics, as we did here. In fact, we conducted state differences detection between EC and EO based on BOLD-ALFF without z-normalization and replicated their findings in the visual cortex (data not shown). Our processing strategy with z-normalization was supported by the finding that standardizing ALFF could reduce the variance induced by nuisance sources such as head motion and different research centers, and improved test-retest reliability [41]. Nevertheless, further investigation is needed to determine the underlying neurophysiologic mechanism of state differences between EC and EO, and whether or not it is appropriate to perform z-normalization on these data.

Lower CBF in the PVC for EC than EO was consistent with a previous ASL study [25] and PET study [42]. Hermes and colleagues [25] acquired resting CBF data using continuous ASL with EC and EO. They showed significant CBF increases in the primary and secondary visual areas (BA 17, 18) for EO compared with EC, and the findings were reproducible on two measurement occasions that were seven weeks apart. Expanding on the temporal reproducibility of state differences between EC and EO detected by CBF, we additionally showed that CBF could consistently detect state differences across research centers.

ASL has also been used to measure dynamic characteristics of CBF [20, 31, 43], though some pitfalls make the dynamic analysis of ASL less reliable than BOLD. These pitfalls include low signal-to-noise ratio, and relatively low temporal resolution (effective TR around 4s for pulsed ASL and longer for pCASL) due to long effective acquisition time. Here we showed that CBF-ALFF demonstrated lower sensitivity to state differences than BOLD-ALFF, possibly because of the lower SNR of CBF dynamics, and highlight the need for further improvements in ASL imaging techniques. It should be noted that CBF-ALFF analysis in this study was only conducted at one research center (NIDA) and the findings needs to be confirmed in future studies.

The neurophysiological interpretation of significant and robust state differences between EO and EC remains challenging. EEG studies have shown that higher power for EC than EO is found in many frequency bands, including delta (1.5–3.5 Hz), theta (4–7.5 Hz), alpha (8–13 Hz), and beta (13.5–25 Hz), over many electrodes [44]. Future studies on EO vs. EC states using BOLD and ASL fMRI, with simultaneous recording of EEG as well as other autonomic measures (e.g., skin conductance, heart rate, and respiration), may help reveal the underlying mechanisms of the state difference between EO and EC.

### Comparison of static and dynamic resting-state activity using BOLD and CBF

The current study showed that there was little overlap of significant between-state differences using static CBF (i.e., CBF-mean) and dynamic BOLD (i.e., BOLF-ALFF) or CBF (i.e., CBF-ALFF), while the overlap between dynamic BOLD and dynamic CBF is greater than the overlap between static and dynamic measures. One likely explanation may be differences in the inherent characteristics of mean CBF and ALFF of BOLD or CBF fluctuations. ALFF measures the strength of the dynamic fluctuations in a time series and therefore primarily captures the temporal characteristics of spontaneous activity. In contrast, mean CBF is a measure of regional blood flow over several minutes, reflecting average cerebral brain metabolism in these regions. Similar phenomena are observed in behavioral neuroscience measurements; for example, reaction time (RT). Traditionally, mean RT has been used as an index for response efficiency, while the low frequency (around 0.05 Hz) fluctuation of intra-individual variability of RT, similar to ALFF of RT (RT-ALFF hereafter), during continuous performance tests (CPT) is a ubiquitous and etiologically important characteristic for children with ADHD [45–48]. The frequency band of RT-ALFF is quite similar to that of resting-state fMRI. However, to our knowledge, no study has investigated the correlation between BOLD-ALFF and RT-ALFF. One study developed a new CPT paradigm, real-time finger force feedback [49] and showed that the ALFF of finger force was negatively correlated with BOLD-ALFF in the precentral gyrus during the continuous feedback task. The correlation between ALFF of behavioral performance and BOLD- or CBF-ALFF during either resting-state or task-state is interesting and could potentially aid our understanding of the underlying relationship between brain and behavior.

Another explanation is the potential difference in flow/metabolism uncoupling [50–52] between task and resting states. Notably, the uncoupling of CBF and CMRO_2_ was revealed in the task state, and it has been postulated that such an uncoupling also occurs in the resting state due to ever-changing spontaneous neuronal activity. It is possible that uncoupling between CBF and oxygen metabolism causes the discrepancy (between mean CBF and strength of BOLD dynamics) observed in this study.

Similarly, no spatial overlap was observed between the state differences detected by static and dynamic characteristics of CBF. This was in agreement with the divergent findings from static CBF and CBF connectivity (though not with CBF-ALFF) when comparing schizophrenic patients and healthy controls [53]. Both the current between-state study and the previous between-population study [53] suggest that CBF dynamics may provide complementary information to static CBF.

### Limitations and future directions

Several limitations should be considered. First, ASL and BOLD acquisitions were not simultaneous, so that optimized scanning parameters for BOLD and ASL contrasts could be applied. In future studies, dual-echo ASL sequence could be used to acquire both contrasts at the same time. In that case, the physiological conditions would be the same for each subject. In addition, using improved ASL techniques with larger effective spatial coverage and higher temporal resolution would allow us to compare the dynamic characteristics of ASL to those of BOLD in different bands across the whole brain. Second, EC and EO were employed as two physiological conditions, and brain activity was compared to reveal the sensitivity of the BOLD and ASL techniques. Future studies might investigate whether the current findings could be extended to other physiological states, e.g., hyperoxia, hypoxia, sleep, and anesthesia. Finally, the current findings should be compared with those yield by a “gold standard” of hemodynamic measurement, e.g., PET.

## Conclusions

The pattern of differences between the EO and EC resting states detected by static activity (CBF-mean of ASL) showed little spatial overlap with that detected by dynamic activity (BOLD- or CBF-ALFF). These results suggest that static CBF and dynamics of BOLD or CBF may reflect different biophysical or physiological processes. Strength of CBF dynamics detected similar state differences to those of BOLD dynamics but with less sensitivity. The combination of ASL and BOLD resting-state fMRI techniques may provide complementary information about the characteristics of resting-state brain activity.
